# Stock market reaction to government policy on determining coal selling price

**DOI:** 10.1016/j.heliyon.2023.e13454

**Published:** 2023-02-08

**Authors:** Sunardi Sunardi, Christiana Noviolla, Supramono Supramono, Yustinus Budi Hermanto

**Affiliations:** a Department of Management, Faculty of Economics and Business, Universitas Merdeka Malang, Indonesia; b EXI Global Aplikasi Indonesia, Indonesia; c Department of Management, Faculty of Economics and Business, Universitas Kristen Satya Wacana, Indonesia; d Economic Faculty of Darma Cendika Catholic University. Management Department, Darma Cendika Catholic University, Jl. Dr. Ir. H. Soekarno No. 201, Surabaya, 60117, East Java, Indonesia

**Keywords:** Domestic market obligation, Coal stock, Abnormal return, Overreaction, Trading volume

## Abstract

The objectives of this study are to analyze the market response by: (a) examining the consequences of the domestic market obligation (DMO) on coal prices policy on the difference in abnormal return (AR) prior to and after the announcement; (b) determine the effect of DMO policy announcements on coal prices on trading volume activity (TVA). This research examined daily stock returns on the shares of 19 coal companies listed on the Stock Exchange in 2018, ten days before and after the DMO announcement (February 23 to March 23, 2018). Statistical analysis was used to calculate the average abnormal return (AAR) and trading volume activity (TVA). The results showed that the announcement of domestic market obligation (DMO) received a negative response from the market. This study also found that the abnormal return was negative eight days before the DMO announcement. This study also finds the cause of overreaction in the short term, namely a significant price reversal process immediately after the announcement of the DMO. The paired sample *t*-test found an insignificant difference in abnormal returns after or before the announcement of the DMO on coal prices policy on companies listed on the IDX for the 2018 period. While testing the TVA, a significant difference was found before and after the announcement of the coal DMO selling price policy.

## Graphical abstract

**Figure:**
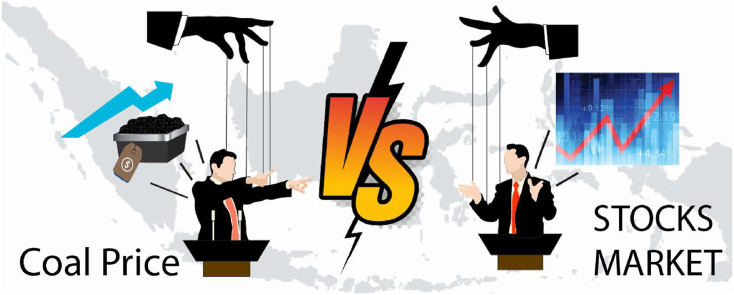

## 1 Introduction

**Fig. 1:**
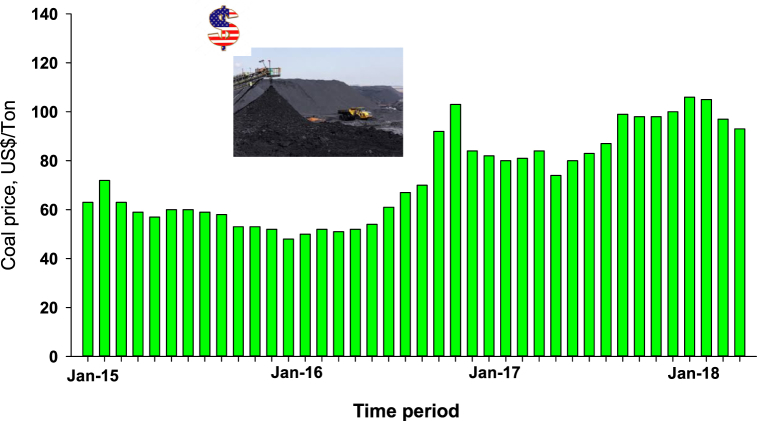
Coal Market Price Fluctuations in 2015–2018 according to the Newcastle Global Coal Index, CV 6322 kcal/kg GAR FOB vessel (in the US $ per tonne) data source: www.iagi.or.id.

A capital market is a place for market participants or investors to invest in financial assets. In investing in the capital market, fluctuating stock price movements can provide advantages and disadvantages for investors. This fluctuating stock price movement can be caused by several factors, one of which is the policy taken by the government [1]. Through Ministerial Decree No. 1395/K/MEM/2018, dated March 9, 2018, the ceiling price for coal is set at USD 70/metric ton and is retroactively valid from January 2018 to December 2019. This price determination results from rising global coal prices [[2], [3], [4]]. The policy on the selling price of coal DMO (Domestic Market Obligation) was made by the government as an act of government protection and supervision over the coal supply for the public interest, especially for the benefit of the electricity fuel supply. Fig. 1 depicts the coal market fluctuation from 2015 to 2018 according to the Newcastle Global Coal Index. Setting the ceiling price of USD 70/metric ton is much lower than the Price Reference Coal (PRC), which reached US $ 101.86 per metric ton on January 2018. This policy was taken to secure the coal supply needed by the state electricity company (PLN) due to rising coal prices reaching USD 100/ton since the second semester of 2016 (Fig. 1). The soaring coal price has caused electricity production costs to increase because 60% of electricity is generated from coal fuel.

The policy that regulates the selling price of Domestic Market Obligation (DMO) coal issued in March 2018 is considered a negative sentiment for investors in the capital market because this policy has pros and cons. On the one hand, this policy positively impacts people's purchasing power, which is maintained because the basic electricity tariff will not fluctuate significantly and impact other sectors. On the other hand, it affects the financial performance of coal companies. It can even impact the market performance of mining companies because the DMO policy can be seen as “bad news” for coal mining companies. The decline in the mining stock index is a form of market reaction in line with the announcement of the DMO policy on coal prices. Coal is a non-renewable natural resource with a limited quantity; currently, coal is still the primary energy source for electricity generation in Indonesia. Most of Indonesia's coal production allocation is exported, and it is increasing yearly. Indonesia's total coal exports were recorded at 390.6 million tons in 2017, an increase of 4.75% from the previous year of 373.9 tons [5]. Although the number of coal reserves is not the largest, Indonesia is the world's largest coal exporter; this is a challenge for the government to better control coal production for energy sustainability/security in the future. Coal also has an essential role in meeting the fuel needs for power generation. The use of coal by PLTU or by PT. PLN and IPP (Independent Power Producer, Private PLTU) constitute the most significant portion of coal consumption each year, an average of 87% of the total realized domestic coal consumption. Coal consumption for electricity needs also increases yearly due to the rapid increase in public electricity consumption.

The empirical study in Pakistan by Mahmood et al. [6] shows a significant relationship between political events and stock market returns. The results of a study conducted by Sari [7] showed that there were significant differences in abnormal return (AR), trading volume activity (TVA), and market capitalization before and after the announcement of information regarding the first reporting of the COVID-19 pandemic in Indonesia. Prasojo [8] studied the stock market reaction to the announcement of the global financial crisis in mining companies. Lestari and Nuzula [9] examined the impact of the Brexit event on AR, and Hill and Maroun [10] examined the effect of violent events that occurred in mining companies in Marikina on stock returns, showing that there is no significant difference in AR between before and after these events.

Meanwhile, the research results of Andita et al. [11] showed a significant difference between AR and TVA before and after the announcement of the third-period tax amnesty policy. Putri [12] analyzed the capital market's reaction before and after the fluctuation of the Rupiah against the US dollar. The Rupiah depreciated the most on March 23, 2020, on the shares of LQ45 index issuers from February 2020 to July 2020. The evidence showed a significant difference in stock AAR and ATVA before the fluctuation of the Rupiah against the US dollar. These results indicate a capital market reaction to the depreciation of the Rupiah against the US dollar in 2020, so it can be concluded that this event contains information for the capital market.

Therefore, analyzing how the market responds to the DMO policy seen from AR and TVA is interesting. Several studies on government policies have been conducted by many researchers, including monetary policy [13,14] and oil price policies [15], and tax reform policies [16]. Studies on the capital market reaction due to tax amnesty policies issued by the Indonesian government [17,18] and economical package policies in 2015–2017 [19] have also been studied by some researchers. However, the previous studies have not analyzed the potential for overreaction, which could lead to prolonged market turmoil due to a government policy. According to De Bondt and Thaler [20], the market can over-respond to information, causing overreaction. This phenomenon of overreaction is an embodiment of market inefficiencies in responding to information.

Previous studies indicate that some government policies cause market reactions, while others have no significant influence on capital markets. Those phenomena underlie this study to re-examine whether government policy announcement events can affect capital market reactions. In our preliminary study [21], we used only AR to measure market efficiency and the effect of DMO on market reaction. This study used both abnormal return (AR) and trading volume activity (TVA) to measure market efficiency and discusses the impact of the announcement of the Domestic Market Obligation (DMO) coal price policy on the market reaction (AR) and TVA. A more comprehensive literature review and discussions are also given in this study. The objectives of this study are to analyze the market response by: (a) examining the consequences of the domestic market obligation (DMO) on coal prices policy on the difference in abnormal return (AR) prior to and after the announcement; (b) determine the effect of DMO policy announcements on coal prices on trading volume activity (TVA). This study provides knowledge about government policies from the market perspective and their impact on potential overreaction. Furthermore, the study's results are expected to inform governments and investors about government policy announcements.

## 2 Literature review

### 2.1 Event study

An event study is a study that examines the reaction of investors in the capital market to the occurrence of an event whose information has been published. The published information can be in the form of 1) information originating from an internal company source (corporate action) that only affects the company's share price; 2) information affecting the prices of securities of several companies. This information can be in the form of government regulations or laws and regulations that affect only the prices of securities of companies subject to those regulations; 3) Information affecting the prices of securities of all companies listed on the capital market. Such information can come from government or regulatory authorities that affect all issuers.

Event studies can also measure the impact of economic events on firm value [22]. Event studies can test two things: the information content of announcements and market efficiency in the semi-strong form. Testing the information content of an announcement aims to see the reaction of investors in the capital market to the announcement. If the announcement contains information, it is expected that investors in the market will react by the time the announcement is received. Changes in the price of the securities in question indicate this investor's reaction.

### 2.2 Market reaction and market efficiency theory

The market will react to events containing information. The event includes an economic value that can change the company's value [23]. An event can be likened to a surprise or something unexpected. The market reaction to an event is proxied with abnormal returns. The greater the surprise, the greater the market reaction. An abnormal return of 0 (zero) indicates that the market did not react to the event. If the market reacts to events, it will obtain a significant abnormal return different from 0 (zero). The sign of a positive or negative abnormal return indicates the direction of the reaction due to a good news event or bad news. Good news events (bad) are expected to be reacted positively (negatively) by the market.

The market is said to be efficient in a semi-strong form if the prices of its securities fully reflect all published information, including information in the issuer company's financial statements. The market is said to be efficient in the strong form if the prices of its securities fully reflect all available information, including private information.

### 2.3 Capital Market Efficiency testing

It is necessary to test to determine the strength and efficiency of a capital market. This test is outlined in a hypothesis referred to as the efficient market hypothesis. Fama [24] divides market efficiency testing into three: 1) Weak form testing, which tests how strongly past price information can predict future returns. Testing is conducted with the return predictability test. 2) Half-strong form testing, which tests how quickly published information is reflected in security prices, is carried out by conducting an event study. 3) Strong form testing, which is to test whether investors who have private information can obtain abnormal returns. The test was carried out using the Test for Private Information.

The more critical the role of the capital market in a country's economy, the more sensitive it is to things that affect it. Events with information content can cause the market to react when they receive information from those events. Events can contain information absorbed by the market and be used by investors, affecting strategy-making or investment decisions. The information can be in the form of non-economic and economic information. These two types of information can affect market reactions; if the information obtained is relevant, it will affect decision-making by investors to get maximum profits. If the market reacts quickly and accurately to reach a new equilibrium price that fully reflects the available information, this market condition is called an efficient market [23]. The capital market is efficient if price information can be obtained openly and quickly without particular obstacles [25]. This information will enter the capital market and then form the price of securities. The market reaction can be seen from the trading volume, price reaction, and reaction to the level of profit (stock returns). Investors need complete, relevant, accurate, and timely information to analyze the capital market.

In an efficient market condition, the market must respond immediately to information so that prices will be formed that reflect the actual condition of the company's fundamentals [24]. Market conditions refer to the assumption that investors act rationally. The information can come from events inside and outside the company [26]. Information from within the company can be in the form of corporate actions, dividend policies, replacement of directors, mergers, and acquisitions. Information from companies can impact the broad market and certain companies or sectors, such as government policies on DMO.

### 2.4 Signaling theory

The market's ability to receive fast and accurate information can be explained through signaling theory. This theory explains that signals originating internally from the company or from external to the company will directly impact the movement of the relevant stock price. Signaling theory is a theory used to understand an action by management in conveying information to investors, which can ultimately change investors' decisions in seeing the company's condition. According to Bringham & Houston [27], the signal theory is an action taken by a company to guide investors about how management assesses the company's prospects. The signal theory explains why the company emphasizes the importance of the information issued to the investment decisions of parties outside the company. Signaling theory is related to information asymmetry; if there is positive information, investors will respond positively and vice versa. Signaling is a unique communication strategy to bridge communication between companies and market participants. Signal theory provides an opportunity to integrate interactive theories of symbolic communication and social benefits with materialist theories of individual strategic action and adaptation [28]. The theory used in this research is the signaling theory and the efficient market hypothesis. The quality of investor decisions is influenced by the company's information disclosed in the financial statements.

### 2.5 The relationship of asymmetric information with Capital Market Efficiency

Asymmetric information, or asymmetric information, is when managers have different (or better) information about the company's future conditions or prospects than those owned by investors [27]. When the information received by investors is different, information asymmetry occurs. Investors who are well informed or know certain information can use this to get abnormal returns. If there is an asymmetry of information, it can be ascertained that the market is in a semi-strong form condition or semi-strong efficiency to allow for abnormal returns.

A study on the relationship of Asymmetric Information to Capital Market Efficiency, conducted by Habib et al. [29], examined the impact of credit rating announcements on the return on shares of 22 banks assessed by the Pakistan Credit Rating Agency and listed on the Karachi Stock Exchange. They showed that the announcement of credit ratings had no significant effect on the abnormal returns of the shares of the sample bank; the downgrade announcement showed a significant positive response, while the upgrade announcement gave an insignificant negative response. The research conducted by Chaudhary et al. [30] on the Karachi Stock Exchange on the event of the announcement of cash dividends on stock returns, and the 15-day event window with the date of the dividend announcement on the day the event was made, showed that the average abnormal returns (AARs), in general, remained positive and statistically significant in the post-event day window. The results of this study support the dividend signaling hypothesis, which suggests that dividend announcements can be used to generate positive signals in the market.

Suryanto [31] conducted a study to analyze stock prices' reaction to Indonesia's announcement of achieving an Investment Grade. The method used is an event study, with a sample of 41 companies on the Indonesia Stock Exchange. Analyze the same price difference using the 11-day window period. The data used are daily stock prices and stock price indices. The results showed a significant difference in abnormal returns the day before the Investment Grade announcement. There was no significant difference in the average abnormal returns before and after the Investment Grade announcement. Another study that made an important contribution to the theory of market efficiency, conducted by Baraccat et al. [32] aimed at analyzing the impact of changes in credit ratings on the long-term returns of Brazilian companies, by conducting an event study to measure how stock prices on the Brazilian Stock Exchange reacted with the appearance of upgrades and downgrades by Moody's and S&P. The results of the study show that stock returns are positive and significant when there is a downgrade and not significant when there is an increase in credit rating.

Asmaranti et al. [33] examined the impact of tick size changes in May 2016 on investor reactions in Indonesia, using t-tests of paired samples to test whether there was a significant difference between investor reactions before and after tick size changes. This study's results show no significant difference between abnormal returns, stock trading transactions, stock trading volume, and risk before and after the event. This study proved that abnormal returns after tick size change events are lower than before. It will be challenging for investors to get a normal profit level consistently. The average stock trading transaction after the event is relatively higher, evidenced by the increased demand for stocks, although the increase is not significant.

The event study conducted by Yoon & Jiang [34] aims to analyze the trends of Chinese companies' revenue–expense matching levels and identify the impact of the 2008 global financial crisis and the decline of the 2015 Shanghai index on this level of matching. The study's results showed that, first, sample analysis for all companies showed that t-1, t, and t+1 expenses significantly positively affected current revenues. Secondly, before and after the 2008 financial crisis, the level of matching changed, but not statistically significant. The implications of empirical results are in the form of accounting policy implications as an analysis of the impact of changes in accounting policies due to macroeconomic events in China on the level of revenue–expense matching.

Investor sentiment is generally based on the theory of financial behavior, is in the trading activities that investors carry out, and can impact the financial markets. Ernawati et al. [35] analyzed investor sentiment toward market reactions on the Indonesia Stock Exchange before and during the Covid-19 pandemic. The proxy of investor sentiment is the trading volume and consumer confidence index, while the market reaction uses the Composite Stock Price Index (CSPI) proxy. Trading volume is a variable that has a positive and significant influence on the CSPI, but the consumer confidence index does not affect the CSPI. During the pandemic, stock prices were at their lowest point, and consumer confidence fell to the point of being pessimistic. On short-selling, irrational investors will tend to make transactions, while their pessimistic investors buy stocks that fall in the hope of higher returns after the pandemic. The results of a study conducted by Wang & Liu [36] on the Chinese stock market showed that the COVID-19 pandemic caused panic in the stock market, which suppressed stock prices and increased volatility in daily returns.

Another empirical study of investor sentiment in stock trading activities was conducted by Chen et al. [37], which investigated the interaction between investor sentiment and the stock market and financial industry by outlining investor sentiment, stock price index, and SWS index of the financial sector. Analysis of the correlation coefficient of time difference is used to determine the medium-and long-term correlation between variables. The analysis results show that investor sentiment significantly correlates with the Shanghai Composite Index, Shenzhen Component Index, and various financial industries represented by the SWS index on its original scale. Changes in investor sentiment are mainly influenced by external market information. The interaction between most markets on a short-term scale is weaker than on its original scale. Investor sentiment is more significantly correlated with SWS Bond, SWS Diversified Finance, and Shanghai Composite Index on the long-term scale than the medium-term scale. Investor sentiment greatly influences the transmission of monetary policy to the stock market. The study of Guo et al. [38] shows that the shock of monetary easing during the sentiment decline phase can increase the stock market by reducing investor fears.

Meanwhile, Huo et al. [39] examined the impact of dual carbon goals on asset prices in China, using President Jinping Xi's speech on September 22, 2020, as an event where the dual carbon goal was officially announced. They found that stocks with a green concept had superior performance in the post-event window compared to non-green stocks. Investors who buy green stocks and sell non-green stocks get an average monthly stock return above 3%. The official announcement of the dual carbon goal caught investors' attention, resulted in higher institutional ownership and trading volumes for green stocks, and impacted improving fundamentals for green stocks in the post-event window.

### 2.6 Stock returns

Stock returns are the results obtained from an investment. Of the various types of returns that will be used in the study of this event is the abnormal return (AR). AR reflects the influence of certain factors of announcements or events, which is why AR is relevant for measuring the market's reaction to the announcement of information. In general, AR is the excess of the actual return on the return expected by investors. According to Jogiyanto [23], an abnormal return is the excess of the return that occurs to the normal return. In this case, the normal return is the return expected by investors. AR is the difference between the actual return minus the expected return. The actual return occurs at time t, which is the difference between the current price and the previous price, while the expected return is the return that is expected to be obtained in the future.

### 2.7 Stock liquidity and trading volume activity

Stock liquidity measures the number of stock transactions in the capital market in a given period. The magnitude of stock liquidity can be measured by calculating the Trading Volume Activity (TVA). Trading Volume Activity **(**TVA) is an increasing function of absolute price changes, where prices reflect the level of information [40]. TVA is an indicator that can be used to see the level of liquidity of stock in the capital market to see how the reaction of the capital market to new information through the parameters of the TVA movement in the market [41]. TVA can be used to see the capital market's reaction to information and the impact of various events.

A stock's trading volume indicates the market's strength or weakness. This concept assumes that an increase or decrease in stock market movements accompanied by large stock trading volumes signifies market power. Investors' reactions in the capital market can be known through changes in stock prices and stock trading volume. Unlike stock prices, stock trading volume is also influenced by the capital market's forces of supply and demand. A stock is declared to have good performance if the trading volume of its shares is high so that the stock is liquid. The fluctuation of stock trading volume for each transaction is a picture of market participants' response to the information in the market [42].

### 2.8 Impact of DMO coal price changes on coal company stock performance

How the market responds to information will have an impact on price changes. If the market responds to information that comes to as good news, then price changes will generate positive returns and vice versa [21]. For example, the market perceives information on some political events [43,44] and terrorism [45,46] as bad news; the market will react negatively. If government policies are considered pro-market, the market will automatically respond positively, and vice versa. For example, the market reacted positively to monetary policy [13] and oil prices [15]. The market responded negatively to the government's financial policy to raise interest rates [14] and Indonesia's economic package policy [19], which also received a negative response from the market. The market responded negatively to the government's policy on coal DMO. This policy is considered to reduce the profits of coal mining companies so that it will reduce the company's performance which will result in a drop in the share price of coal mining companies. This phenomenon can be seen from the negative abnormal returns on the days before and at the time of the announcement of the DMO [21].

The dominance of the type of investor will significantly affect the stock price movement toward the DMO announcement. If investors move quickly and excessively when information is received, there will be a price reversal or overreaction, and vice versa [21]. In the capital market, there are two types of investors: rational and irrational. Rational investors are investors who are free from negative sentiments, while irrational investors are investors who tend to have negative sentiments [47]. If irrational investors dominate a capital market, information will be handled excessively; the market will become inefficient because stock prices become too high or too low [21]. The concept of overreaction was first introduced by Kahneman and Tversky [48]. Their psychological research proved that dramatic and unexpected events cause humans to experience overreactions. In a capital market, often information considered good will cause investors to exaggerate stock prices. Investors tend to undervalue the stock if the information received is not good [49]. Husaini [50] also found that in the capital market, good or bad news tends to cause investors to overreact. Investor psychology is also very influential in overreaction in the capital market [[51], [52], [53]]. If investors realize they have overreacted, their correction can cause a price reversal [54]. The same phenomenon regarding price reversal because investors know they have overreacted also found in several studies [49,54,55]. Investors' overreaction in the capital market can occur over a long or short time [56,57]. As an example, Smith [58] analyzed Dow stock returns to normal within a short period of 10 days. Investors also overreacted when the government announced a policy that the selling price of DMO coal would be far below the HBA price. For investors, this policy is bad news [21].

The existence of the DMO policy, as stated in Government Regulation No. 08 of 2018, which is an amendment to Government Regulation No. 23 of 2010 concerning the Implementation of Mineral and Coal Mining Business Activities, causes the mining index to decrease, causing an abnormal return [59]. This is expected to provide a bad signal or news for capital market players because this policy will reduce profits from coal companies. After the announcement of the government's plan to limit the price of DMO coal, within two days, the market capitalization value of coal issuers on the Indonesia Stock Exchange (IDX) decreased from Rp11.7 to Rp11.4 trillion [59]. With this event, the allegations that foreign and domestic investors made a stronger sell-off. The efficient capital market hypothesis states that an efficient market reacts quickly to relevant information [60]. The sooner new information is reflected in the price of a security, the more efficient the capital market will be. In an efficient market, the market will react quickly and accurately to incoming new information to achieve a new equilibrium price that fully reflects the available information. In an efficient market, there is no possibility of obtaining an abnormal rate of return, although, in practice, there are distorted things called anomalies. The market's ability to receive fast and accurate information can be explained through signaling theory. This theory explains that signals from internal and external companies will directly impact stock price movements. Based on the previous description, the hypothesis in this study is formulated as follows.

H1 There is a significant difference in abnormal returns (AR) before and after the announcement of the coal DMO selling price policy for coal companies listed on the Indonesia Stock Exchange (IDX) for 2018.

H2 There is a significant difference in trading volume activity (TVA) before and after the announcement of the coal DMO selling price policy for coal companies listed on the Indonesia Stock Exchange (IDX) for 2018.

## 3 Research methodology

### 3.1 Research design

This study analyzes market movements and trades that are affected by an event, so the methodology used to detect market responses to events that are reflected in changes in stock prices is the event study methodology [21,22]. The event used in this research is government policy which regulates DMO coal prices on March 9, 2018. This event study analyzed market reactions to government policies regarding coal DMO [21,23]. In this study, the benchmarks used to prove the market reaction to changes in DMO coal prices were indicators of abnormal returns and trading volume activity.

The market reactions were measured by looking at changes in abnormal returns. The event study analyzed the abnormal returns of securities that occurred around the announcement of the event study. Abnormal return is the excess of the actual return that occurs to the normal return. If an announcement containing information will give an abnormal return to the market, if it does not include information, it will not provide AR. In addition, the event study also analyzes the volume of stock trading. A stock's trading volume indicates the market's strength or weakness. This concept assumes that an increase or decrease in stock market movements accompanied by large stock trading volumes signifies market strength. In contrast, if relatively large volumes do not accompany it, it indicates a weak market.

The event chosen in this study is the DMO announcement on March 9, 2018, regarding the selling price of coal for domestic use (maximum US$70). This price is below the HBA price, which is bad news for companies in the coal sector and can cause negative abnormal returns. This study uses Yahoo Finance [61] data, corporate action data, and company IPO obtained from IDX's official website [62].

### 3.2 Population and samples

The coal companies that were the object of this research were listed on the Indonesia Stock Exchange (IDX) in 2018 (Table 1). The following criteria were used to choose the samples.

(a) The company's shares were actively traded during the study period from December 4, 2017, to March 23, 2018.
(b) To avoid the confounding effect, the coal companies that are the object of research do not carry out corporate actions during the observation period, such as stock splits, dividend announcements, rights issues, mergers, and others.
(c) The coal company that was the object of research has been listed on the stock exchange at least one year before the government regulation regarding coal DMO. There were 27 companies listed on the Indonesian stock exchange, 6 company shares were not actively traded, and 2 shares were listed on the stock exchange one month before the government regulation, so only 19 companies met the requirements (Table 1).

**Table 1:** Research samples.

No	Stock Code	Issuer Name
1	ADRO	Adaro Energy Tbk
2	ARII	Atlas Resources Tbk
3	BSSR	Baramulti Suksessarana Tbk
4	BUMI	Bumi Resources Tbk
5	BYAN	Bayan Resources Tbk
6	DEWA	Darma Henwa Tbk
7	DOID	Delta Dunia Makmur Tbk
8	FIRE	Alfa Energi Investama Tbk
9	GTBO	Garda Tujuh Buana Tbk
10	HRUM	Harum Energy Tbk
11	ITMG	Indo Tambangraya Megah Tbk
12	KKGI	Resources Alam Indonesia Tbk
13	MBAP	Mitrabara Adiperdana Tbk
14	MYOH	Samindo Resources Tbk
15	PKPK	Perdana Karya Perkasa Tbk
16	PTBA	Tambang Batubara Bukit Asam (Persero) Tbk
17	PTRO	Petrosea Tbk
18	SMMT	Golden Eagle Energy Tbk
19	TOBA	Toba Bara Sejahtera Tbk

### 3.3 Analysis techniques

The observation period was determined for 21 days for the data analysis stage, ten days each before and after the government's announcement of the coal DMO (23 February-23 March 2018). This study used the method of Spyrou et al. [63], with an estimated 50-day period (t-60, t-11), which started 60 days and ended ten days before the event day, to avoid the possibility of price increases before the event.

The following steps were used to determine abnormal returns and Trading Volume Activity.

a. Abnormal Return

Abnormal return is the difference between the actual return minus the expected return. The model used to calculate the research data was market-adjusted, based on events that were not previously estimated and had no estimation period. The stages of calculation and the equations used (Equations (1), (2), (3), (4), (5))) to calculate abnormal returns are as follows.

1) Determine the actual return in the window period

Actual return is used as the basis for calculating the return on expectations, the equation used is as follows:

(1) $$R_{i,t}=\frac{\left(P_{t}-P_{t-1}\right)}{P_{t-1}}$$

Where:

*R*_*i, t*_: Stock Return for Securities to -i on -t day.

*P*_*t*_: The price of the stock i in the period t.

*P*_*t-1*_: The price of the stock i in the period _t-1._

2) Determine the Market-Adjusted Model

Market index return is the difference between today's closing market index and the previous day divided by the last day's closing market index, and use the following equation:

(2) $$E\left[R_{i,t}\right]=\frac{R_{Mi.t}-R_{Mi.t-1}}{R_{Mi.t-1}}$$

Where:

*E [R*_*i,t*_*]***:** Expected Return. Return of a market index (market adjusted model) at the estimated period t.

*R*_*Mi.t*_: Market return from stock to -i in the period of the event to -t.

3) Abnormal Return

The following equation calculates abnormal returns:

(3) $$AR_{i,t}=R_{i,t}-E\left[R_{i,t}\right]$$

Where:

*AR*_*i.t*_: abnormal return for securities to -i on day -t.

*R*_*i,t*_: Actual return for the stock to -i on event period to -t.

*E [R*_*i,t*_*]*: Market index return (market return) at the estimated period t.

4) Average Abnormal Return (AAR)

The formula for calculating the AAR is as follows:

(4) $$AAR_{t}=\sum_{t=i}^{k}\frac{AR_{i,t}}{n}$$

Where:

*AAR*_*t*_: Average Abnormal Return on day -t.

*AR*_*i.t*_: Abnormal return for securities to -i on day -t.

*N*: The number of companies used as samples.

After the average abnormal return data is obtained, the data tabulation is carried out. Furthermore, a hypothesis test was carried out using the One-Sample *t*-Test statistical test. If the value sig.≥ α, i.e., 0.05, then hypothesis 1 is rejected, while if the value of sig. < α, then hypothesis 1 is accepted.

5) Analyzing the possibility of overreaction by detecting a reversal through a cumulative average abnormal return (CAAR) graph and a positive and significant AAR value.

(5) $$CAAR_{t}=\frac{\sum AAR_{t}}{n}$$

Where:

*CAAR*_*t*_ = Average Abnormal Return in period t.

*AAR*_*t*_ = average abnormal return period t

*N* = number of observation periods.

b. Trading Volume Activity (TVA)

1) Calculation of Trading Volume Activity (TVA)

TVA is obtained by comparing the number of issuers traded in a certain period with the total number of issuers outstanding in the same period. Equation (6) is used to determine *TVA*_***it***_:

(6) $${TVA}_{it}=\frac{The\;volume\;of\;stock\;traded\;at\;time\;t}{The\;volume\;of\;stock\;i\;outstanding\;in\;the\;period\;t}$$

Where:

TVA_it:_ Trading Volume Activity for stocks to – i on day to -t.

2) Average Trading Volume Activity (ATVA)

TVA in this study was analyzed using Average Trading Volume Activity (ATVA) in each company. The first step is to conduct a descriptive analysis of TVA by dividing the average trading volume into two groups, ATVA, before and after announcing the coal DMO selling price policy. This descriptive analysis also adds trading volume data at the time of the coal DMO selling price policy announcement so that trading volume can be observed at the time of the announcement. Equation (7) is used to calculate the value of ATVA before and after the announcement of the coal DMO selling price policy:

(7) $$ATVA_{t}=\frac{\sum TVA_{t}}{n}$$

where:

*ATVA*_*t*_ = Average trading volume activity on day - t.

*TVA* = number of company TVA

*N* = number of observation periods.

After tabulating the data, a normality test was carried out using the Kolmogorov-Smirnov statistical test. Subsequently, the hypothesis is tested using the Independent Sample *t*-Test if the data is normally distributed. The Independent sample *t*-test is a test to determine the presence or absence of significant differences in the average ATVA before and after the event.

## 4 Analysis and discussion

### 4.1 Description of AAR

Table 2 shows the movement of AAR 10 days before to after the DMO announcement, which shows the abnormal return value that occurred in the event period. If observed, at t-10 to t-9, subsequently at t-7 to t-3, no significant abnormal returns were found due to sig values. (2-tailed) greater than 0.05. This indicates that from February 25, 2018, to March 4, 2018, the market has not reacted to the DMO event. On t-2 and t-1, the market responded to the event because there was a significant abnormal return with a significance level of less than 0.05.

**Table 2:** Significance of AAR during the observation period.

t	AAR	CAAR	*t*-test	(Sig)
t-10	0.002	0.002	0.499	0.623
t-9	−0.003	−0.003	−1.876	0.076
t-8	−0.013	−0.013	−3.249	0.004^a^
t-7	−0.011	−0.011	0.603	0.553
t-6	−0.014	−0.014	−0.429	0.629
t-5	−0.0474	0.001	−0.270	0.788
t-4	−0.0009	−0.0483	−1.009	0.867
t-3	−0.0132	−0.0615	−0.170	0.076
t-2	−0.0113	−0.0729	−1.882	0.017^a^
t-1	0.0194	−0.0535	4.228	0.001^a^
t0	−0.0049	−0.0583	−0.576	0.572
t1	−0.0171	−0.0755	−2.279	0.035^a^
t2	0.002	−0.0734	−0.383	0.706
t3	−0.0006	−0.074	−0.0840	0.934
t4	−0.0093	−0.0833	−1.488	0.154
t5	−0.0171	−0.1004	−2.614	0.018^a^
t6	−0.054	−0.054	1.612	0.123
t7	−0.057	−0.057	−1.411	0.174
t8	−0.041	−0.041	2.508	0.021^a^
t9	−0.041	−0.041	−0.036	0.972
t10	−0.038	−0.038	0.894	0.383

a Note: Significance at 0.05.

On t0 day, which is on March 7, 2018, President Jokowi signed Government Regulation No. 08 of 2018 regarding the DMO Coal Price regulation; there is no significant abnormal rate of return. This happened because some investors did not react to the signing issue. However, on t1, t5, and t8, the market reacted again to the event, where t1 is the day before the policy was published, investors began to react and think about the impact that would occur on the mining company if the policy took effect because it could reduce the company's profit. On t2 to t4, there is also no significant abnormal return because it has a sig value > 0.05. It was not until t-1, which was the day before the signing of PP No. 08 of 2018 that the AAR was found to be of positive and significant value. However, the average abnormal return on the other few days is negative (although not all are significant). This means that Government Regulation No. 08 of 2018 regarding the regulation on reducing DMO Coal Prices is bad news for market participants.

**Fig. 2:**
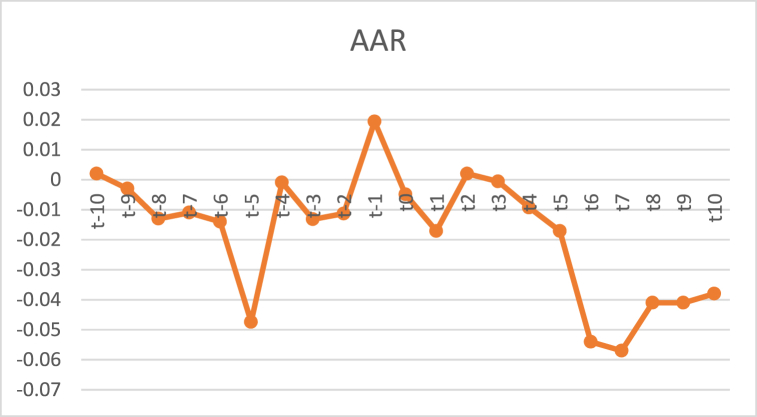
Average abnormal return.

Changes in AAR values for 21 days can be seen in Fig. 2. Before the announcement, AAR was negative on t-9 to t-2 and had experienced a positive increase in t-1 and t2, and AAR experienced subtraction on t3 to t7, then began to increase on t8.

The results of the normality test based on Kolmogorov-Smirnov (0.140, sign = 0.112) and Shapiro Wilk (0.948, sign 0.316) are higher than the significance value > 0.05, indicating that the data is normally distributed.

### 4.2 Market response regarding DMO announcement seen from AAR

Testing the market response to the DMO announcement, which is proxied by the value of AAR, using a one-sample *t*-test as presented in Table 2. Significant negative AAR occurred at t-8, t-2, t-1, t1, t5, and t8. This shows the negative reaction given by the market to the DMO announcement made by the Indonesian government.

In January 2018, the Minister of Energy and Mineral Resources informed the public that he was considering a new scheme to include coal prices as a reference in setting the basic electricity tariff in 2018. At the end of February 2018, the Ministry of Energy and Mineral Resources seeks to set coal prices for domestic electricity needs*.* Based on the tirto. id newspaper on March 13, 2018, this new policy revised the timing of implementing the reference coal price regulation for domestic purposes (domestic market obligation/DMO), which was originally retroactive from January 1, 2018, to December 2019. With the revised policy announced on March 9, 2018, investors still felt uncertain about this policy.

It is interesting to analyze that significant negative AAR has occurred since eight days before the announcement of the DMO regulating the selling price of coal. This shows that the announcement responded negatively and suspected that information leakage had occurred or the market had taken anticipative action before the rules on knowing coal prices were announced on March 9, 2018. Market participants digest this information by interpreting that the mining sector is currently in a weak state, and automatically, most investors decide to sell their shares, causing a negative AR. The difference in AAR of the 20 coal companies sampled before and after the announcement of the coal DMO selling price policy indicates that the policy announcement contains information that makes the capital market react.

### 4.3 Overreaction DMO announcement seen from CAAR

**Fig. 3:**
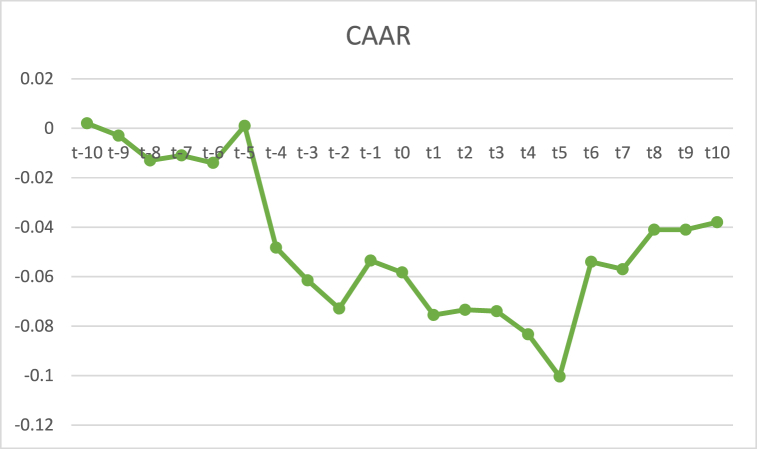
Cumulative average abnormal return.

It is necessary to analyze whether there is a positive and significant CAAR around the DMO announcement to indicate a price reversal process due to an overreaction. In Table 2, it can be seen that the CAAR is significantly positive at t8 (sign = 0.021). These results can also be seen through the CAAR graph in Fig. 3 after the announcement of the DMO. The price reversal process started at t4, t5, and t6, where prices increased even though at t7 it had experienced a slight decrease, but only at t8 the price experience a significant increase.

DMO announcements are considered bad news and cause an excessive market response. This is in line with De Bondt and Thaler's [20] research and Fang's [50], which found that market participants overestimate prices when they receive bad information, so investors tend to sell shares excessively and cause an unnatural price decrease. Significant negative reactions after the events only occurred on the second to the third day indicate that the market only reacted for a moment or short term, and the next day the stock price began to rise. These results align with Nam, Pyun & Avard's [57] research, which found that overreaction can occur quickly.

### 4.4 AAR difference test before and after announcement of coal DMO selling price policy

This test was conducted to determine the difference between 2 samples interconnected with the assumption of normal data distribution, namely the difference in AAR before and after the announcement of the coal DMO selling price policy, using α = 0.05. The test results can be seen in the following table.

Table 3 shows that from a sample of 19 companies (n) that have a significance value (Asymp. Sig. 2-tailed) of 0.241 > 0.05, this indicates that H1 is rejected, which means there is no significant difference in abnormal returns before and after the policy announcement. The selling price of coal DMO on coal companies listed on the Indonesia Stock Exchange (IDX) for 2018. Fig. 2 shows that although after the announcement of the coal DMO selling price policy, there was a sharp decrease in AAR at t7, starting at t8, AAR began to move up compared to the decline in AAR before the policy announcement (t-5), and the selling price of coal DMO was lower. The results of this study indicate that the announcement of the coal DMO selling price policy does not provide a significant difference to the AAR value of the mining subsector companies listed on the IDX for the 2018 period. The absence of a significant abnormal difference in returns before and after the announcement of the DMO selling price policy is indicated because the company's returns are not large enough that they cannot form a significant difference. This is possible because the coal DMO selling price policy is timing differences which means that the decline in stock prices occurs due to uncertainty-circulating related to the DMO policy. Hence, investors hold back their investment in coal companies. This is because the discourse on the coal DMO selling price policy has been launched since early 2018, so information regarding the announcement of the coal DMO selling price was known to investors before the policy was announced. The leak of this information made investors react in advance to minimize losses that might occur due to the announcement of the policy. Mixed market reactions influence the difference in returns that have not changed significantly. Investors argue that this regulation is not an extraordinary event that should be feared because it has been promulgated, so mining companies carry out much anticipation to maintain the sustainability of their business. Apart from regulations that make it easier for mining companies to make adjustments, the government also continues to allow the export of raw goods to companies that have already entered into agreements. The government also imposes progressive exit tariffs to reduce the potential for loss or misuse of permits to continue exports. This policy is to dampen the desire of mining companies to export massively before the deadline for banning all mining goods and raw minerals, as described in the relevant law is officially enforced.

**Table 3:** Wilcoxon signed range AAR test results before and after announcement of coal DMO selling price policy.

Pair 1	Z	−1.56
AARt-n	N	19
AAR + n	Asymp. Sig. (2-tailed)	0.241

The results align with the study by Sari [7] (2021), which shows a significant abnormal return on one day around the event date, which means that the market reacts to the event. This study's results align with Asmaranti et al. [33], which showed no significant difference in the abnormal return on stocks in Indonesia before and after the tick size change event in May 2016. Likewise, Suryanto's study [31] on 41 companies on the Indonesian stock exchange showed a significant abnormal difference in returns the day before the announcement of the Investment Grade. Still, there was no significant difference in the average abnormal return before and after the announcement of the Investment Grade. The study by Baraccat et al. [32] also showed no significant difference in stock returns when there was an increase in credit rating on the Brazilian stock exchange.

The results of this study are not in line with the signaling theory, which reveals that every action (event) can provide a signal to the market in the form of a positive or a negative sign. If an event contains essential information, it will cause the capital market to react, which is seen from the AAR value. The study results are also in line with Prasojo [8], who studied the stock market reaction to the announcement of the global financial crisis in mining companies. Lestari and Nuzula [9], who examined the impact of the Brexit event on AR; and Hill and Maroun [10], who studied the effect of violent events that occurred in mining companies in Marikan on stock returns, which showed that there was no significant difference in AR between before and after these events.

### 4.5 Market reaction regarding DMO announcement seen from ATVA

The following are the results of ATVA calculations before and after announcing the coal DMO selling price policy for 19 coal companies listed on the IDX for 2018.

Table 4 shows that the standard deviation of ATVA has decreased from 129, 656, 477.09 before the announcement of the coal DMO selling price policy to 95, 319, 898.75 after the announcement of the policy. This indicates that the ATVA before the policy announcement was more diverse than the ATVA after the coal DMO selling price policy announcement.

**Table 4:** ATVA before, during, and after announcement of coal DMO selling price policy.

	TVA (in units of shares)		
	ATVA Before	ATVA at the time	ATVA After
Amount	1,019,762,718.43	721,117,100	701,827,754.57
Lowest Value	4342.86	1700	1764.86
The highest Value	468,023,285.71	286,969,100	408,500,300
Average	53,671,722.02	37,953,531.58	36,938,302.87
Standard Deviation	129,656,477.09	75,589,584.90	95,319,898.75

### 4.6 ATVA difference test before and after announcement of coal DMO selling price policy

This test was conducted to determine the correlation and difference between TVA before and after announcing the policy on coal DMO selling prices, using α = 0.05. The test results are shown in Table 5.

**Table 5:** ATVA paired samples correlations test results before and after announcement of coal DMO selling price policy.

Pair 1	ATVA Before-After Announcement
N	19
Correlation	0.995
Sig.	0.000

The paired sample correlations test (Table 6) shows that the correlation value is 0.995 with a significance value of 0.000 < 0.05, indicating a significant relationship between the average data groups. The test results indicate an important relationship between ATVA before and after announcing the coal DMO selling price policy.

**Table 6:** ATVA Paired Sample *t*-Test Results Before and After Announcement of Coal DMO Selling Price Policy.

		Pair 1 TVA before–after the announcement
Paired Differences	Mean	0.381
	Std. Deviation	0.582
	Std. Error Mean	0.134
	95% Confidence Lower	0.100
	Internal of the Uppe	0.662
t		2.871
Df		19
Sig. (2-tailed)		0.01

Table 6 shows the positive statistical *t*-test is 2.871 (higher than 2.000) with a significance value of 0.01 < 0.05. These results indicate that the ATVA before the announcement is higher than after the policy announcement, and H2 is accepted. This means there is a significant difference in trading volume activity before and after the policy announcement. The significant difference in trading volume activity between before and after the announcement of the coal DMO selling price policy occurred because the number of shares traded in the period around the announcement of the policy decreased. This condition is caused by investors selling shares of coal issuers as a preventive action to minimize losses. The findings support signaling theory [64] so that recipients of information or the market will respond to information obtained from management and the mass media as signals of certain events that can affect value. The study results align with Andita et al. [11], which showed a significant difference in the ATVA before and after the announcement of the third-period tax amnesty policy. Likewise, the results of Putri's research [12] showed a significant difference in ATVA before and after the fluctuation of the Rupiah against the US dollar.

The results of other studies support the efficient market theory conducted by Ernawati et al. [35]. They studied market sentiment as measured by the volume of stock transactions before and after the Covid-19 pandemic on the stock market's reaction to the Indonesian stock exchange. Before the Covid-19 pandemic, investor sentiment did not affect market reactions; on the contrary, during the Covid-19 pandemic, investor sentiment did influence market reactions. The study of Chen et al. [37] shows that investor sentiment changes are more influenced by external market information. The efficient market theory states that investors cannot use publicly available information, such as historical stock price or trading volume, to find excess returns in the stock market [60]. An understanding of how the behavioral characteristics of investors affect the mechanism of information transmission in the money market and capital market prices needs to be improved; and essential to the decision-making of financial analysts and investors by asking companies to significantly improve the information environment in good times and bad [65].

## 5 Conclusion

This study analyzes the market reaction and overreaction to the DMO announcement, which regulates the selling price of coal for domestic interests at US $ 70 per metric ton, far below the HBA price of US $ 101.86 per ton. The study results show that the DMO announcement is considered a bad news market that responds negatively to the prices of shares of companies engaged in the Coal sector. In addition, the market also overreacted, marked by negative abnormal returns around the day of the event. However, it is short-term, as evidenced by a price reversal that occurred on the eighth day after the announcement. However, the paired sample *t*-test results show no significant difference in abnormal returns before and after the announcement of the coal DMO selling price policy on coal issuers listed on the IDX for the 2018 period. This is because the company's stock price during the observation period is insufficient. Large enough to cause a difference in returns before and after the coal DMO selling price policy announcement.

The results of the paired sample *t*-test on trading volume activity show a significant difference between before and after the announcement of the coal DMO selling price policy on coal issuers listed on the IDX for the 2018 period. This is because changes in the trading volume of companies during the observation period are relatively large, causing differences in trading volume activity between before and after the announcement of the coal DMO selling price policy issued on March 9, 2018.

The limitation of this study is that it only tests the information content of an event and uses trading volume to measure the level of liquidity of the stocks around the event. For further study, it is necessary to pay attention to the following points.

1). The research should not only examine the information from an event but also proceed with testing market efficiency. Event studies can not only be used as a test of the information contained in an event but also as an initial stage to test market efficiency.
2). Using return expectations by comparing calculation models, namely the mean, market, and market-adjusted models. The results of such comparisons can be used as a consistent comparison tool for observing research results.
3) Further study needs to consider other factors besides trading volume, for example, the frequency of stock trading transactions.

The results of this study show that investors can already be said to be intelligent investors because they are not easily panicked by the policies issued by the government. Investors understand the purpose and objectives of a policy and can implement good decisions related to their investments. Suppose a policy like this is issued later by the government. In that case, companies and investors do not need to worry about the impact of a policy because the community has enough time to understand the benefits and objectives of the policy. Many parties will benefit when a policy has been disseminated long before its implementation. Apart from the fact that investors are given time to adjust their funds' allocation, rule-makers can also minimize the negative impact related to the shock caused by the realization of a policy. Parties directly related to the new policy (companies) will also be able to prepare themselves in various aspects better. The impacts resulting from policies that have long been socialized will be far more effective and accepted by the public because they do not cause significant panic. This study shows a substantial difference in stock trading, but stock returns do not show a significant difference.

Academically reinforce empirical evidence that the market does not always respond to an event or announcement only when the event occurs, leading to inefficient markets, as stated by De Bondt & Thaler [48]. The research uses abnormal returns and trading volume to measure the market reaction that occurs; it is hoped that further research will use additional indicators to describe the market reaction that occurs more broadly. Further research could create a longer event window after the event as there is still a significant abnormal return after five days. That allows abnormal returns to continue for a longer period. The longer event window allows the researchers to know the number of days of the market reaction to the announcement of the coal DMO price policy more precisely.

Based on the behavioral finance approach, overreaction occurs because transaction decisions tend to be influenced by psychological factors rather than corporate funds. In practical terms, policymakers should prioritize fair policy-making and conduct public communication before a policy is issued so that it does not cause market shock and even cause overreaction. As for investors, it is advisable not to rush to sell action against government policies because, based on research results, it is proven that overreaction is only short-term. Coal issuers must be more careful in looking at the trend of the coal trading market and global reference coal prices. The alternative is to overcome the coal DMO price policy on the company's performance by looking at the worldwide reference coal price fluctuations. The decline in international reference coal prices to lower than the selling price of coal DMO (<USD 70/mt) can be used by coal issuers to reduce exports, potentially reducing the decline in prices for coal issuers decline in globally reference coal prices. For investors in the capital market in general, it is hoped that they can more quickly and efficiently utilize any information published by the company to get maximum profit.
